# Tris(diisopropyl di­thio­phosphato-κ^2^ *S*,*S*′)ruthenium(III)

**DOI:** 10.1107/S1600536813014141

**Published:** 2013-05-25

**Authors:** Guo-Ping Chao, Xiuli Wu, Hua-Tian Shi, Qun Chen, Qian-Feng Zhang

**Affiliations:** a Department of Applied Chemistry, School of Petrochemical Engineering, Changzhou University, Jiangsu 213164, People’s Republic of China; b Institute of Molecular Engineering and Applied Chemsitry, Anhui University of Technology, Ma’anshan, Anhui 243002, People’s Republic of China

## Abstract

In the title complex, [Ru(C_6_H_14_O_2_PS_2_)_3_], the coordination environment of the Ru^III^ atom is distorted octa­hedral, defined by six S atoms from three *S*,*S*′-bidentate diisopropyl di­thio­phosphate ligands. The average Ru—S bond length is 2.41 (1) Å and the average S—Ru—S bite angle is 81.13 (19)°.

## Related literature

For background to ruthenium complexes, see: Castillo-Villalón *et al.* (2008 ▶); Chianelli *et al.* (2009 ▶); David *et al.* (2005 ▶); Leung *et al.* (2000 ▶); Wu *et al.* (2009 ▶). For related structures, see: Jain *et al.* (2000 ▶); Liu *et al.* (2005 ▶).
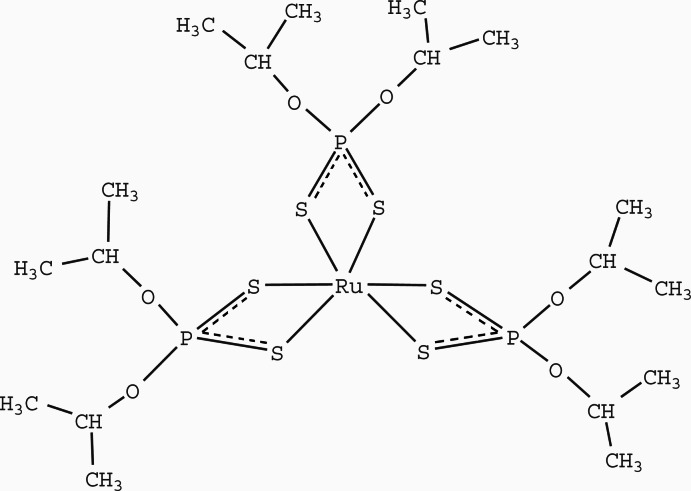


## Experimental

### 

#### Crystal data

[Ru(C_6_H_14_O_2_PS_2_)_3_]
*M* *_r_* = 740.92
Triclinic, 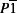

*a* = 8.9676 (8) Å
*b* = 10.5073 (9) Å
*c* = 19.1085 (17) Å
α = 81.281 (2)°
β = 88.678 (2)°
γ = 82.175 (2)°
*V* = 1763.1 (3) Å^3^
*Z* = 2
Mo *K*α radiation
μ = 0.96 mm^−1^
*T* = 296 K
0.14 × 0.11 × 0.10 mm

#### Data collection

Bruker APEXII CCD diffractometer
Absorption correction: multi-scan (*SADABS*; Sheldrick, 1996 ▶) *T*_min_ = 0.877, *T*_max_ = 0.910
11411 measured reflections
7478 independent reflections
6083 reflections with *I* > 2σ(*I*)
*R*_int_ = 0.022

#### Refinement

*R*[*F* ^2^ > 2σ(*F* ^2^)] = 0.032
*wR*(*F* ^2^) = 0.083
*S* = 1.02
7478 reflections
319 parameters
H-atom parameters constrained
Δρ_max_ = 0.47 e Å^−3^
Δρ_min_ = −0.32 e Å^−3^

### 

Data collection: *APEX2* (Bruker, 2007 ▶); cell refinement: *SAINT* (Bruker, 2007 ▶); data reduction: *SAINT*; program(s) used to solve structure: *SHELXS97* (Sheldrick, 2008 ▶); program(s) used to refine structure: *SHELXL97* (Sheldrick, 2008 ▶); molecular graphics: *SHELXTL* (Sheldrick, 2008 ▶); software used to prepare material for publication: *SHELXTL*.

## Supplementary Material

Crystal structure: contains datablock(s) I, global. DOI: 10.1107/S1600536813014141/hy2626sup1.cif

Structure factors: contains datablock(s) I. DOI: 10.1107/S1600536813014141/hy2626Isup2.hkl

Additional supplementary materials:  crystallographic information; 3D view; checkCIF report

## Figures and Tables

**Table 1:** Selected bond lengths (Å)

Ru1—S1	2.4189 (7)
Ru1—S2	2.4037 (7)
Ru1—S3	2.3981 (7)
Ru1—S4	2.3988 (7)
Ru1—S5	2.4155 (7)
Ru1—S6	2.4199 (7)

**Fig. 1.**
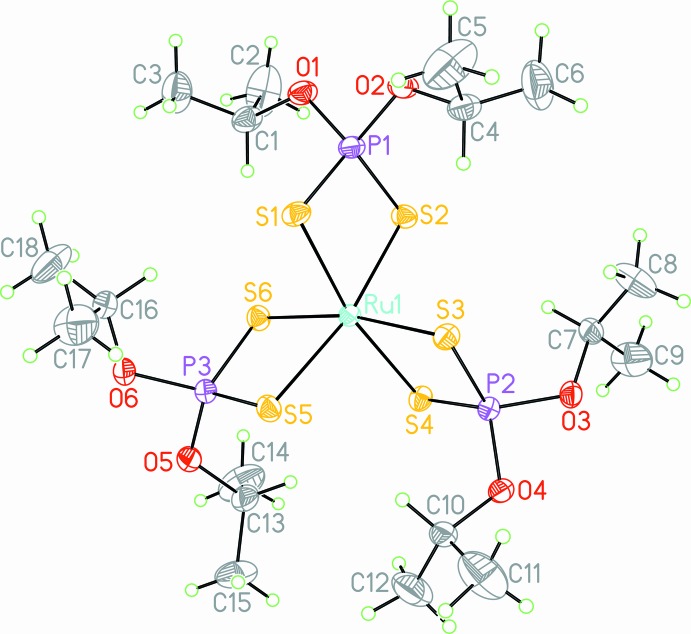
The molecular structure of the title compound. Displacement ellipsoids are drawn at the 50% probability level.

## supplementary crystallographic information

### Comment

In recent years there has been an increased interest in ruthenium complexes with sulfur-donor ligands, in part because of the high catalytic activity of RuS_2_ unit in various hydrogenation processes (Castillo-Villalón *et al.*, 2008; Chianelli *et al.*, 2009). In the course of our continuous study on ruthenium complexes in a sulfur-rich coordination environment (Leung *et al.*, 2000), we are interested in the homoleptic ruthenium complexes with thiolate ligands, which may be probably designed as processors for the binary RuS_2_ nanoparticles (David *et al.*, 2005). Although the ruthenium chemistry of dithio acidic ligands such as dithiocarbamate and dithiocarbonate has been the subject of continuous study, the corresponding ruthenium dithiophosphate chemistry has not been developed much (Wu *et al.*, 2009). Here we report the crystal structure of the title compound, a homoleptic ruthenium complex.

The molecular structure of the title complex is depicted in Fig. 1. The complex is mononuclear and the Ru^III^ atom displays a distorted octahedral RuS_6_ coordination geometry. Each of dithiophosphate ligands binds to the Ru^III^ atom in an S,S'-bidentate mode, forming a four-membered ring with an average S—Ru—S bite angle of 81.31 (2)°, which is comparable with those in [Ru{S_2_P(OMe)_2_}_3_] [av. 81.54 (8)°] and [Ru{S_2_P(OEt)_2_}_3_] [av. 81.84 (6)°] (Jain *et al.*, 2000). Each four-membered RuS_2_P ring is nonplanar and contains a pair of nearly equal Ru—S bonds (Table 1). The average Ru—S bond length of 2.4092 (7) Å in the title complex is compatible to those in [Ru{S_2_P(OMe)_2_}_3_] [av. 2.413 (13) Å] and [Ru{S_2_P(OEt)_2_}_3_] [av. 2.424 (3) Å] (Jain *et al.*, 2000), but is obviously shorter than those in [Ru{S_2_P(OEt)_2_}_2_(PPh_3_)_2_] [av. 2.4974 (11) Å] and [RuH(CO){S_2_P(OEt)_2_}(PPh_3_)_2_] [av. 2.5474 (12) Å] (Liu *et al.*, 2005). The bond distances within the di-*iso*-proposaldithiophosphate ligands of the title complex agree well with those found in the analogous dimethyl- and diethyldithiophosphate complexes of ruthenium (Jain *et al.*, 2000).

### Experimental

A mixture of RuCl_3_.H_2_O (209 mg, 0.80 mmol) and KS_2_P(O*^i^*Pr)_2_ (606 mg, 2.40 mmol) was dissolved in 25 ml of methanol and then heated at reflux for 8 h. During this time the color of the reaction solution was changed from brown to bright red. The solvent was evaporated in vacuo and the residue was redissolved in dichloromethane and then filtered. The filtrate was dried and then recrystallized from diethyl ether/hexane. The red plate-shaped crystals of the title complex were obtained within a week. Yield: 260 mg, 44% (based on Ru). Analysis, calculated for C_18_H_42_O_6_P_3_RuS_6_: C 29.18, H 5.71%; found: C 29.25, H 5.67%.

### Refinement

H atoms were placed in geometrically idealized positions and refined as riding atoms, with C—H = 0.98 (CH) and 0.96 (CH_3_) Å and with *U*_iso_(H) = 1.2(1.5 for methyl)*U*_eq_(C).

### Crystal data

**Table:**

[Ru(C_6_H_14_O_2_PS_2_)_3_]	*Z* = 2
*M**_r_* = 740.92	*F*(000) = 766
Triclinic, *P*1	*D*_x_ = 1.396 Mg m^−^^3^
Hall symbol: -P 1	Mo *K*α radiation, λ = 0.71073 Å
*a* = 8.9676 (8) Å	Cell parameters from 2994 reflections
*b* = 10.5073 (9) Å	θ = 2.6–25.7°
*c* = 19.1085 (17) Å	µ = 0.96 mm^−^^1^
α = 81.281 (2)°	*T* = 296 K
β = 88.678 (2)°	Block, red
γ = 82.175 (2)°	0.14 × 0.11 × 0.10 mm
*V* = 1763.1 (3) Å^3^	

### Data collection

**Table:**

Bruker APEXII CCD diffractometer	7478 independent reflections
Radiation source: fine-focus sealed tube	6083 reflections with *I* > 2σ(*I*)
Graphite monochromator	*R*_int_ = 0.022
φ and ω scans	θ_max_ = 27.2°, θ_min_ = 2.1°
Absorption correction: multi-scan (*SADABS*; Sheldrick, 1996)	*h* = −11→11
*T*_min_ = 0.877, *T*_max_ = 0.910	*k* = −13→12
11411 measured reflections	*l* = −24→17

### Refinement

**Table:**

Refinement on *F*^2^	Primary atom site location: structure-invariant direct methods
Least-squares matrix: full	Secondary atom site location: difference Fourier map
*R*[*F*^2^ > 2σ(*F*^2^)] = 0.032	Hydrogen site location: inferred from neighbouring sites
*wR*(*F*^2^) = 0.083	H-atom parameters constrained
*S* = 1.02	*w* = 1/[σ^2^(*F*_o_^2^) + (0.0341*P*)^2^ + 0.2818*P*] where *P* = (*F*_o_^2^ + 2*F*_c_^2^)/3
7478 reflections	(Δ/σ)_max_ = 0.001
319 parameters	Δρ_max_ = 0.47 e Å^−^^3^
0 restraints	Δρ_min_ = −0.32 e Å^−^^3^

### Special details

**Table:**

Geometry. All esds (except the esd in the dihedral angle between two l.s. planes) are estimated using the full covariance matrix. The cell esds are taken into account individually in the estimation of esds in distances, angles and torsion angles; correlations between esds in cell parameters are only used when they are defined by crystal symmetry. An approximate (isotropic) treatment of cell esds is used for estimating esds involving l.s. planes.
Refinement. Refinement of F^2^ against ALL reflections. The weighted R-factor wR and goodness of fit S are based on F^2^, conventional R-factors R are based on F, with F set to zero for negative F^2^. The threshold expression of F^2^ > 2sigma(F^2^) is used only for calculating R-factors(gt) etc. and is not relevant to the choice of reflections for refinement. R-factors based on F^2^ are statistically about twice as large as those based on F, and R- factors based on ALL data will be even larger.

### Fractional atomic coordinates and isotropic or equivalent isotropic displacement parameters (Å^2^)

**Table:**

	*x*	*y*	*z*	*U*_iso_*/*U*_eq_	
Ru1	0.73359 (2)	0.556490 (18)	0.733889 (10)	0.04029 (7)	
S1	0.76352 (8)	0.32294 (7)	0.76690 (4)	0.05468 (17)	
S2	0.94238 (8)	0.50082 (7)	0.65811 (4)	0.05370 (17)	
S3	0.53460 (8)	0.54659 (6)	0.65399 (4)	0.05063 (16)	
S4	0.70241 (8)	0.77884 (6)	0.67808 (4)	0.04961 (16)	
S5	0.55622 (7)	0.59086 (7)	0.82845 (4)	0.05446 (17)	
S6	0.90545 (7)	0.59914 (7)	0.82065 (4)	0.05280 (17)	
P1	0.93826 (8)	0.31504 (7)	0.69980 (4)	0.05098 (17)	
P2	0.53435 (7)	0.73678 (6)	0.62205 (3)	0.04477 (15)	
P3	0.72941 (8)	0.61841 (7)	0.88599 (4)	0.05070 (17)	
O1	1.0904 (2)	0.2439 (2)	0.73416 (11)	0.0644 (5)	
O2	0.9293 (2)	0.2225 (2)	0.64323 (11)	0.0645 (5)	
O3	0.5505 (2)	0.7768 (2)	0.54005 (9)	0.0563 (5)	
O4	0.37794 (19)	0.82026 (18)	0.62947 (9)	0.0551 (5)	
O5	0.7094 (2)	0.74961 (19)	0.91720 (10)	0.0644 (5)	
O6	0.7469 (2)	0.5263 (2)	0.95902 (10)	0.0624 (5)	
C1	1.1586 (4)	0.2919 (3)	0.79194 (19)	0.0748 (9)	
H1	1.0995	0.3727	0.8019	0.090*	
C2	1.3140 (5)	0.3157 (6)	0.7684 (3)	0.151 (2)	
H2A	1.3645	0.2406	0.7505	0.226*	
H2B	1.3691	0.3321	0.8078	0.226*	
H2C	1.3080	0.3896	0.7318	0.226*	
C3	1.1601 (5)	0.1891 (5)	0.8553 (2)	0.1150 (16)	
H3A	1.0598	0.1685	0.8648	0.172*	
H3B	1.1968	0.2201	0.8955	0.172*	
H3C	1.2246	0.1126	0.8463	0.172*	
C4	0.7981 (4)	0.2394 (3)	0.5962 (2)	0.0735 (9)	
H4	0.7245	0.3110	0.6079	0.088*	
C5	0.7314 (6)	0.1163 (5)	0.6098 (4)	0.167 (3)	
H5A	0.8043	0.0460	0.5992	0.251*	
H5B	0.6445	0.1231	0.5804	0.251*	
H5C	0.7024	0.1001	0.6587	0.251*	
C6	0.8515 (5)	0.2709 (6)	0.5218 (2)	0.149 (2)	
H6A	0.9104	0.3414	0.5185	0.224*	
H6B	0.7664	0.2956	0.4908	0.224*	
H6C	0.9121	0.1960	0.5082	0.224*	
C7	0.6915 (3)	0.7423 (3)	0.50207 (15)	0.0623 (8)	
H7	0.7729	0.7080	0.5359	0.075*	
C8	0.6647 (6)	0.6420 (5)	0.4587 (3)	0.1266 (18)	
H8A	0.6194	0.5746	0.4875	0.190*	
H8B	0.7588	0.6057	0.4402	0.190*	
H8C	0.5987	0.6809	0.4202	0.190*	
C9	0.7253 (5)	0.8642 (4)	0.4591 (3)	0.1215 (17)	
H9A	0.6431	0.8988	0.4274	0.182*	
H9B	0.8155	0.8469	0.4322	0.182*	
H9C	0.7391	0.9261	0.4897	0.182*	
C10	0.2862 (4)	0.8074 (3)	0.69364 (16)	0.0675 (8)	
H10	0.3235	0.7271	0.7249	0.081*	
C11	0.1293 (5)	0.8026 (6)	0.6708 (3)	0.141 (2)	
H11A	0.0966	0.8784	0.6373	0.212*	
H11B	0.0637	0.8000	0.7113	0.212*	
H11C	0.1267	0.7262	0.6492	0.212*	
C12	0.2940 (5)	0.9204 (5)	0.7298 (3)	0.1266 (18)	
H12A	0.3974	0.9317	0.7349	0.190*	
H12B	0.2481	0.9063	0.7757	0.190*	
H12C	0.2417	0.9968	0.7023	0.190*	
C13	0.6915 (4)	0.8748 (3)	0.87079 (19)	0.0766 (9)	
H13	0.6763	0.8600	0.8222	0.092*	
C14	0.8314 (6)	0.9343 (5)	0.8733 (3)	0.148 (2)	
H14A	0.8432	0.9553	0.9199	0.222*	
H14B	0.8255	1.0120	0.8393	0.222*	
H14C	0.9160	0.8741	0.8625	0.222*	
C15	0.5537 (5)	0.9537 (5)	0.8945 (3)	0.1347 (19)	
H15A	0.4684	0.9083	0.8921	0.202*	
H15B	0.5371	1.0360	0.8642	0.202*	
H15C	0.5668	0.9676	0.9423	0.202*	
C16	0.7732 (4)	0.3847 (3)	0.96155 (16)	0.0659 (8)	
H16	0.7969	0.3654	0.9136	0.079*	
C17	0.6338 (5)	0.3299 (5)	0.9858 (3)	0.1299 (18)	
H17A	0.6092	0.3480	1.0328	0.195*	
H17B	0.6492	0.2376	0.9861	0.195*	
H17C	0.5527	0.3685	0.9544	0.195*	
C18	0.9062 (5)	0.3339 (5)	1.0075 (3)	0.138 (2)	
H18A	0.9910	0.3754	0.9893	0.207*	
H18B	0.9292	0.2417	1.0081	0.207*	
H18C	0.8843	0.3516	1.0547	0.207*	

### Atomic displacement parameters (Å^2^)

**Table:**

	*U*^11^	*U*^22^	*U*^33^	*U*^12^	*U*^13^	*U*^23^
Ru1	0.04333 (12)	0.03643 (11)	0.04021 (12)	−0.00469 (8)	0.00048 (8)	−0.00357 (8)
S1	0.0558 (4)	0.0391 (4)	0.0658 (4)	−0.0044 (3)	0.0055 (3)	0.0006 (3)
S2	0.0540 (4)	0.0504 (4)	0.0555 (4)	−0.0055 (3)	0.0117 (3)	−0.0071 (3)
S3	0.0542 (4)	0.0429 (4)	0.0563 (4)	−0.0088 (3)	−0.0084 (3)	−0.0091 (3)
S4	0.0585 (4)	0.0386 (3)	0.0517 (4)	−0.0116 (3)	−0.0064 (3)	−0.0013 (3)
S5	0.0491 (4)	0.0671 (5)	0.0463 (4)	−0.0058 (3)	0.0055 (3)	−0.0083 (3)
S6	0.0497 (4)	0.0619 (4)	0.0478 (4)	−0.0078 (3)	−0.0042 (3)	−0.0107 (3)
P1	0.0486 (4)	0.0443 (4)	0.0592 (4)	0.0025 (3)	−0.0037 (3)	−0.0123 (3)
P2	0.0481 (4)	0.0438 (4)	0.0402 (3)	−0.0021 (3)	−0.0014 (3)	−0.0030 (3)
P3	0.0603 (4)	0.0498 (4)	0.0400 (4)	−0.0007 (3)	−0.0017 (3)	−0.0061 (3)
O1	0.0550 (11)	0.0601 (13)	0.0764 (14)	0.0102 (9)	−0.0149 (10)	−0.0183 (10)
O2	0.0603 (11)	0.0594 (13)	0.0755 (14)	0.0092 (9)	−0.0128 (10)	−0.0292 (10)
O3	0.0535 (10)	0.0707 (13)	0.0399 (10)	0.0003 (9)	0.0003 (8)	−0.0008 (8)
O4	0.0541 (10)	0.0539 (11)	0.0507 (10)	0.0060 (8)	0.0039 (8)	0.0005 (8)
O5	0.0869 (14)	0.0532 (12)	0.0512 (11)	0.0021 (10)	−0.0025 (10)	−0.0116 (9)
O6	0.0852 (14)	0.0580 (12)	0.0409 (10)	−0.0009 (10)	−0.0023 (9)	−0.0052 (8)
C1	0.069 (2)	0.068 (2)	0.087 (2)	0.0054 (16)	−0.0263 (18)	−0.0192 (18)
C2	0.122 (4)	0.197 (6)	0.139 (5)	−0.095 (4)	−0.040 (3)	0.029 (4)
C3	0.108 (3)	0.148 (5)	0.082 (3)	−0.012 (3)	−0.015 (2)	0.002 (3)
C4	0.0653 (19)	0.062 (2)	0.095 (3)	0.0054 (15)	−0.0227 (18)	−0.0271 (18)
C5	0.167 (5)	0.085 (3)	0.257 (7)	−0.045 (3)	−0.107 (5)	−0.007 (4)
C6	0.118 (4)	0.250 (8)	0.081 (3)	−0.007 (4)	−0.022 (3)	−0.041 (4)
C7	0.0580 (16)	0.077 (2)	0.0490 (16)	0.0018 (15)	0.0072 (13)	−0.0101 (14)
C8	0.140 (4)	0.133 (4)	0.124 (4)	−0.028 (3)	0.050 (3)	−0.075 (3)
C9	0.122 (3)	0.104 (4)	0.126 (4)	−0.015 (3)	0.063 (3)	0.009 (3)
C10	0.076 (2)	0.0612 (19)	0.0558 (17)	0.0121 (16)	0.0166 (15)	0.0008 (14)
C11	0.100 (3)	0.206 (6)	0.143 (5)	−0.072 (4)	0.053 (3)	−0.068 (4)
C12	0.131 (4)	0.146 (5)	0.114 (4)	−0.004 (3)	0.020 (3)	−0.072 (3)
C13	0.103 (3)	0.0522 (19)	0.071 (2)	0.0045 (18)	−0.0121 (19)	−0.0085 (15)
C14	0.132 (4)	0.073 (3)	0.232 (7)	−0.026 (3)	−0.018 (4)	0.013 (4)
C15	0.142 (4)	0.085 (3)	0.163 (5)	0.037 (3)	0.005 (4)	−0.021 (3)
C16	0.083 (2)	0.0575 (19)	0.0513 (17)	0.0013 (16)	0.0020 (15)	0.0002 (13)
C17	0.126 (4)	0.092 (3)	0.163 (5)	−0.028 (3)	0.040 (3)	0.012 (3)
C18	0.164 (4)	0.082 (3)	0.155 (5)	0.013 (3)	−0.083 (4)	0.007 (3)

### Geometric parameters (Å, º)

**Table:**

Ru1—S1	2.4189 (7)	C6—H6A	0.9600
Ru1—S2	2.4037 (7)	C6—H6B	0.9600
Ru1—S3	2.3981 (7)	C6—H6C	0.9600
Ru1—S4	2.3988 (7)	C7—C9	1.477 (5)
Ru1—S5	2.4155 (7)	C7—C8	1.483 (5)
Ru1—S6	2.4199 (7)	C7—H7	0.9800
S1—P1	2.0027 (10)	C8—H8A	0.9600
S2—P1	1.9983 (10)	C8—H8B	0.9600
S3—P2	1.9988 (9)	C8—H8C	0.9600
S4—P2	2.0030 (9)	C9—H9A	0.9600
S5—P3	2.0007 (10)	C9—H9B	0.9600
S6—P3	1.9976 (10)	C9—H9C	0.9600
P1—O2	1.570 (2)	C10—C12	1.471 (5)
P1—O1	1.5706 (19)	C10—C11	1.493 (5)
P2—O4	1.5653 (18)	C10—H10	0.9800
P2—O3	1.5681 (18)	C11—H11A	0.9600
P3—O5	1.570 (2)	C11—H11B	0.9600
P3—O6	1.5709 (19)	C11—H11C	0.9600
O1—C1	1.459 (4)	C12—H12A	0.9600
O2—C4	1.472 (4)	C12—H12B	0.9600
O3—C7	1.473 (3)	C12—H12C	0.9600
O4—C10	1.460 (3)	C13—C14	1.480 (5)
O5—C13	1.462 (4)	C13—C15	1.493 (5)
O6—C16	1.468 (4)	C13—H13	0.9800
C1—C3	1.494 (5)	C14—H14A	0.9600
C1—C2	1.496 (5)	C14—H14B	0.9600
C1—H1	0.9800	C14—H14C	0.9600
C2—H2A	0.9600	C15—H15A	0.9600
C2—H2B	0.9600	C15—H15B	0.9600
C2—H2C	0.9600	C15—H15C	0.9600
C3—H3A	0.9600	C16—C17	1.483 (5)
C3—H3B	0.9600	C16—C18	1.487 (5)
C3—H3C	0.9600	C16—H16	0.9800
C4—C5	1.483 (5)	C17—H17A	0.9600
C4—C6	1.494 (5)	C17—H17B	0.9600
C4—H4	0.9800	C17—H17C	0.9600
C5—H5A	0.9600	C18—H18A	0.9600
C5—H5B	0.9600	C18—H18B	0.9600
C5—H5C	0.9600	C18—H18C	0.9600
			
S3—Ru1—S4	81.50 (2)	H6A—C6—H6B	109.5
S3—Ru1—S2	98.00 (3)	C4—C6—H6C	109.5
S4—Ru1—S2	91.82 (2)	H6A—C6—H6C	109.5
S3—Ru1—S5	91.52 (3)	H6B—C6—H6C	109.5
S4—Ru1—S5	95.44 (3)	O3—C7—C9	105.9 (3)
S2—Ru1—S5	168.80 (3)	O3—C7—C8	107.4 (3)
S3—Ru1—S1	90.71 (3)	C9—C7—C8	113.0 (4)
S4—Ru1—S1	168.84 (3)	O3—C7—H7	110.1
S2—Ru1—S1	81.31 (2)	C9—C7—H7	110.1
S5—Ru1—S1	92.73 (3)	C8—C7—H7	110.1
S3—Ru1—S6	169.79 (2)	C7—C8—H8A	109.5
S4—Ru1—S6	92.10 (2)	C7—C8—H8B	109.5
S2—Ru1—S6	90.11 (3)	H8A—C8—H8B	109.5
S5—Ru1—S6	81.12 (3)	C7—C8—H8C	109.5
S1—Ru1—S6	96.67 (3)	H8A—C8—H8C	109.5
P1—S1—Ru1	87.31 (3)	H8B—C8—H8C	109.5
P1—S2—Ru1	87.83 (3)	C7—C9—H9A	109.5
P2—S3—Ru1	87.78 (3)	C7—C9—H9B	109.5
P2—S4—Ru1	87.67 (3)	H9A—C9—H9B	109.5
P3—S5—Ru1	87.61 (3)	C7—C9—H9C	109.5
P3—S6—Ru1	87.56 (3)	H9A—C9—H9C	109.5
O2—P1—O1	96.41 (11)	H9B—C9—H9C	109.5
O2—P1—S2	113.86 (9)	O4—C10—C12	108.7 (3)
O1—P1—S2	114.71 (9)	O4—C10—C11	106.8 (3)
O2—P1—S1	114.42 (9)	C12—C10—C11	111.2 (4)
O1—P1—S1	114.49 (9)	O4—C10—H10	110.0
S2—P1—S1	103.50 (4)	C12—C10—H10	110.0
O4—P2—O3	96.25 (10)	C11—C10—H10	110.0
O4—P2—S3	113.93 (8)	C10—C11—H11A	109.5
O3—P2—S3	114.89 (9)	C10—C11—H11B	109.5
O4—P2—S4	115.64 (8)	H11A—C11—H11B	109.5
O3—P2—S4	113.79 (8)	C10—C11—H11C	109.5
S3—P2—S4	102.97 (4)	H11A—C11—H11C	109.5
O5—P3—O6	96.44 (11)	H11B—C11—H11C	109.5
O5—P3—S6	113.64 (9)	C10—C12—H12A	109.5
O6—P3—S6	114.96 (9)	C10—C12—H12B	109.5
O5—P3—S5	115.12 (9)	H12A—C12—H12B	109.5
O6—P3—S5	113.51 (9)	C10—C12—H12C	109.5
S6—P3—S5	103.70 (4)	H12A—C12—H12C	109.5
C1—O1—P1	121.08 (19)	H12B—C12—H12C	109.5
C4—O2—P1	120.73 (18)	O5—C13—C14	108.5 (3)
C7—O3—P2	122.02 (16)	O5—C13—C15	107.3 (3)
C10—O4—P2	123.28 (17)	C14—C13—C15	114.4 (4)
C13—O5—P3	121.11 (19)	O5—C13—H13	108.8
C16—O6—P3	120.41 (17)	C14—C13—H13	108.8
O1—C1—C3	107.1 (3)	C15—C13—H13	108.8
O1—C1—C2	106.9 (3)	C13—C14—H14A	109.5
C3—C1—C2	112.1 (3)	C13—C14—H14B	109.5
O1—C1—H1	110.2	H14A—C14—H14B	109.5
C3—C1—H1	110.2	C13—C14—H14C	109.5
C2—C1—H1	110.2	H14A—C14—H14C	109.5
C1—C2—H2A	109.5	H14B—C14—H14C	109.5
C1—C2—H2B	109.5	C13—C15—H15A	109.5
H2A—C2—H2B	109.5	C13—C15—H15B	109.5
C1—C2—H2C	109.5	H15A—C15—H15B	109.5
H2A—C2—H2C	109.5	C13—C15—H15C	109.5
H2B—C2—H2C	109.5	H15A—C15—H15C	109.5
C1—C3—H3A	109.5	H15B—C15—H15C	109.5
C1—C3—H3B	109.5	O6—C16—C17	109.1 (3)
H3A—C3—H3B	109.5	O6—C16—C18	107.7 (3)
C1—C3—H3C	109.5	C17—C16—C18	114.2 (3)
H3A—C3—H3C	109.5	O6—C16—H16	108.6
H3B—C3—H3C	109.5	C17—C16—H16	108.6
O2—C4—C5	106.5 (3)	C18—C16—H16	108.6
O2—C4—C6	107.9 (3)	C16—C17—H17A	109.5
C5—C4—C6	113.4 (4)	C16—C17—H17B	109.5
O2—C4—H4	109.6	H17A—C17—H17B	109.5
C5—C4—H4	109.6	C16—C17—H17C	109.5
C6—C4—H4	109.6	H17A—C17—H17C	109.5
C4—C5—H5A	109.5	H17B—C17—H17C	109.5
C4—C5—H5B	109.5	C16—C18—H18A	109.5
H5A—C5—H5B	109.5	C16—C18—H18B	109.5
C4—C5—H5C	109.5	H18A—C18—H18B	109.5
H5A—C5—H5C	109.5	C16—C18—H18C	109.5
H5B—C5—H5C	109.5	H18A—C18—H18C	109.5
C4—C6—H6A	109.5	H18B—C18—H18C	109.5
C4—C6—H6B	109.5		

## References

[bb1] Bruker (2007). *APEX2* and *SAINT* Bruker AXS Inc., Madison, Wisconsin, USA.

[bb2] Castillo-Villalón, P., Ramírez, J. & Maugé, F. (2008). *J. Catal.* **260**, 65–74.

[bb3] Chianelli, R. R., Berhault, G. & Torres, B. (2009). *Catal. Today*, **147**, 275–286.

[bb4] David, D., Silvia, E. & Castillo, B. (2005). *J. Phys. Chem. B*, **109**, 22715–22724.

[bb5] Jain, P. U., Munshi, P., Walawalkar, M. G., Rath, S. P., Rajak, K. K. & Lahiri, G. K. (2000). *Polyhedron*, **19**, 801–808.

[bb6] Leung, W. H., Lau, K. K., Zhang, Q. F., Wong, W. T. & Tang, B. (2000). *Organometallics*, **19**, 2084–2089.

[bb7] Liu, X., Zhang, Q. F. & Leung, W. H. (2005). *J. Coord. Chem.* **58**, 1299–1305.

[bb8] Sheldrick, G. M. (1996). *SADABS* University of Göttingen, Germany.

[bb9] Sheldrick, G. M. (2008). *Acta Cryst.* A**64**, 112–122. 10.1107/S0108767307043930 18156677

[bb10] Wu, F. H., Duan, T., Lu, L., Zhang, Q. F. & Leung, W. H. (2009). *J. Organomet. Chem.* **694**, 3844–3851.

